
JOURNAL INFORMATION
==============================
NLM Title Abbreviation: Med Chir Rev
NLM Journal ID: 0034617

The Medico-Chirurgical Review

Publisher: Biomedical Journal Digitization Project

ARTICLE INFORMATION
==============================
PMCID: PMC4966265
PMID: 29917635
Article version: 1
Subjects: Reviews, Diseases Produced by Malaria

An Essay on Remittent and Intermittent Diseases, Including Marsh Fever, Neuralgia, Tic Douloureux, Sciatica, &c.

Print publication date: 1828 Oct 1
Volume: 9
Issue: 18
First page: 289
Last page: 307
1828 Jul
License: This work is free of known copyright restrictions. For more information, please see PMC Back Issue Digitization.
License URL: https://creativecommons.org/publicdomain/mark/1.0/

==============================

THE

### Medico-Chirurgical Review,

No. XVIII.

JULY, AUGUST, SEPTEMBER, 1828.

### I.

### DISEASES PRODUCED BY MALARIA.

*An Essay on the Remittent and Intermittent Diseases, including Marsh Fever, Neuralgia, Dysentery, Cholera, Tic Douloureux, Sciatica, Head-ache, Palsy, &c. &c.* By John Macculloch, M.D. F.R.S.

[Art. II.]

DYSENTERY—CHOLERA—AGUE.

In pursuance of our design to exhibit an extensive survey of our ingenious author's researches, we naturally turn from remittent fever to dysentery, cholera, and ague. This last malady is now very generally prevalent over England, though masked and disguised in a thousand different shapes. We, therefore, intreat a patient and very careful perusal of the following article, and we beg that each practitioner will look round him, with an unprejudiced eye, for proofs or refutations of the views here taken. This exercise of his intellects will be amply repaid in the end.

In respect to the first of these scourges of the human race, although Dr. Macculloch has but little personal experience, he has ample materials to draw upon in support of his doctrines, from the experience of others. Very few authors have written on dysentery, without tracing its cause, especially when epidemic or endemic, to terrestrial or aerial influence—aerial *impregnations* being as often blamed as atmospheric *vicissitudes*. The connexion of this disease with intermittent and remittent fevers has been remarked by all writers. The *nature* of this connexion, that is, the *proximate cause*, or condition of dysentery, is rather timidly, if not reluctantly, touched on by Dr. M.

"To illustrate, slenderly, what is here meant, we have, in severe cases of the remittent fever, that local affection of the stomach which causes the black vomit, that analogous one of the liver which produces what are called bilious symptoms, affections of the head, and so forth, besides all the more rigidly local and partial diseases of which I have here treated at some length. It is not therefore difficult to comprehend, yet very generally and broadly, how such localization, or determination to the intestines, may produce dysentery; while this will vary as fever is conspicuously combined with it, or as the local affection is such as to supersede in a great degree the general one: while yet further, it is easy to imagine that if the affection in question should attach preferably to one or to another portion of the intestines, to the duodenum for example in one case and to the colon in another, (a fact very conceivable from their differences in character, structure, or sensibility,) all the varieties of dysentery as to the most obvious effects might be the result." Vol. I. p. 218.

This view, Dr. M. observes, becomes nearly identical with "that French theory, often differing from former ones only in its terms," which supposes dysentery to be an enteritis, *i.e.* inflammation of the mucous membrane of the small or great intestines. The difficulty, as Dr. M. justly remarks, lies in accounting for the morbific miasm or malaria producing in one man a remittent, in a second, an intermittent, and, in a third, dysentery. Our author conceives that there is little, if any, real distinction between diarrhœa and dysentery. He evidently believes that the late endemic at the Penitentiary was the product of malaria, a doctrine, by the way, which is not impugned by the succeeding healthiness of the place. In all parts of the world, we see localities healthy for a series of years, and then suddenly, and without any ostensible cause, becoming completely pestilential. The East and West Indies, the Netherlands—in short, all parts of Europe furnish abundant examples. Dysentery, in this kingdom, is seldom severe or dangerous, and its treatment is pretty well understood. In tropical climates, where the disease rages on a larger scale, medical practitioners are also pretty much of one opinion as to the treatment.

In respect to cholera, we have always maintained that it is the product of some deleterious agent, emanating from the earth, and more or less diffused in the air. Dr. M. has come to the same conclusion. The following is one of the facts which have led him to this conclusion.

"A frigate had cruised long on the coasts of India where this disease was raging on shore, retaining her health, however, and, as was believed, by rigidly avoiding any communication with, or even approach to the land; the captain's conduct in this respect having arisen from his experience of African fevers. Being on one occasion at anchor about three miles from the shore, the land wind came off to the vessel, and with such effect, that the usual smell attending Malaria, well known to the officers and men from their African experience, was immediately sensible. There was not at this time a sick man on board, while there had been no communication with the land for many weeks: and the alarm being immediately taken, the vessel was ordered to weigh for sea; while, as had been the constant practice, every man not wanted on deck, was ordered below, for the purpose of avoiding the effect of the Malaria thus blown off to sea in the manner which I have described in the essay on that subject. An accident having happened to the iron cable, the armourer was the first man employed, almost alone, on deck, in disengaging it; and though in perfect health when he came up, he was immediately seized with giddiness, was quickly rendered incapable of proceeding with his work, became insensible within three hours, and died of this cholera, which also seized on four of the crew before the vessel could get under weigh; the whole of them dying in the same manner." 222.

Dr. M. conjectures that the Indian cholera results from the ordinary miasma of fever in some peculiarly concentrated state, the effects bearing an analogy to what sometimes happens in the plague. The local affections of the stomach, bowels, and liver, are the most prominent features in this dire disease—we might therefore, Dr. M. thinks, consider cholera as a "localized remittent of peculiar severity, and with two or three local affections at one time"—"a fever, in which minutes perform the destructive duty of hours, days, or even weeks; or in which, from its rapidity, there is scarcely an initial stage—as if the disorder commenced where it more usually terminates." With other conjectures, or plausible hypotheses respecting cholera, we shall not trouble our readers, at the present time, but proceed to the next subject.

### INTERMITTENT FEVER.

We agree with the author, that there is hardly a fact better established in medicine, than that ague is produced by malaria. He thinks it is by no means proved that there is any other cause—at least of the original disease, or first attack, although various causes may have the power of re-exciting the disease. We shall not follow Dr. M. through a detail of the common phenomena or symptoms of ague:—they have, of late years, been presented pretty generally even to English practitioners. But we shall here notice some very interesting observations which our author has made on certain states of mind attendant on, or connected with intermittents, whether obvious or obscure in their forms. This state of mind is a peculiar irritability or despondency, more especially revealing itself in the cold stage, or incipient movements of the disease.

"It is in fact at times, the sole cold fit, or almost the only disease; though a watchful eye may always discover that it is connected with that collapse of features or change of expression which attends every cold fit of intermittent, and, very commonly, with that peculiar physiognomy, easier recognized than explained, which, to an observant eye, is always sufficient to indicate every disease, general or local, connected with intermittent, or arising from Malaria; an appearance which, when more strongly marked in the pallid hue of the face and the shrinking of the nose, ought to decide the question, even to the most negligent observer.

"Of its true nature and cause, be the moral results what they may, no doubt can be entertained, because of the suddenness of the attack, and of its periodical character; and it thus happens that in almost a second of time, and even in the midst of active good humour, or passive feelings of comfort or happiness, the fit of ill temper, or irritability, or despair, of a moral change under modes too various to detail, will occur, to last as long as the cold fit would have lasted had it been present; or lasting, when that is visible, just as long as the duration of the peculiar physiognomy which I have described. To be aware of this fact and this cause, on the part of the patient's circle, is to be furnished with reasons for making that charitable excuse which is seldom made, if ever, for what self-control might be supposed capable of preventing, and perhaps not much oftener for what is unavoidable; so rare is this species of charity: for the patient to be aware, himself, of the cause, is to furnish him with a guide for his own conduct in these circumstances, and a check over the display of those feelings; while it is also to offer him the consolation of knowing that it is his body rather than his mind which is diseased; that his perverted temper is not a moral and voluntary fault or failing; but as involuntary as it is capable of being remedied." 246.

Such a disorder of mind may be readily confounded with hypochondriacism. Such are the milder moral derangements in the milder chronic intermittents.

"But in severe cases of both, the united state of irritability and despair is apt to produce the far more serious effects of stimulating the patient, at least to think of suicide. This insane desire is a very common complaint of patients labouring under intermittent, and a very frequent source of great alarm and horror; while it occurs equally in patients who, before that, were cheerful, as well as youthful, in the female sex as in the male, in persons where, either from previous knowledge of their opinions and characters, or from observation in the absence of the fit, we are quite sure that it cannot depend on a wrong state of mind or of opinions, but is as rigidly a portion of the disease as it is found to be a periodical one." 248.

That such states of mind are very frequently produced by corporeal derangements we well know—and that these bodily affections are not seldom dependent on the impression or reception of malaria, we have strong reason to believe. How generally malaria deranges the functions of the viscera, and especially the liver, stomach, and other digestive organs, need not now be told; and that these derangements produce the most dreadful mental despondency, and lead often to suicide, are facts that are every day becoming more obvious to the attentive medical observer.

It is very important that these effects of intermittents should be known and understood both by patient and practitioner—since a knowledge of the real causes will be a great relief to the minds of those who, under such circumstances, are haunted for years with this species of phrensy or mental alienation. The desire for suicide is often exceedingly temporary, even when it is most overpowering; and if controlled with resolution, it soon passes away. To be aware, therefore, of the real nature of the adumbration may frequently tend to obviate its dire effects.

"There is another remark yet, which it may be worth while to make on this mental disease, whether or not it is really connected with intermittent in all cases. I have clearly ascertained it to be so, at least in some; or rather, every instance of the desire or attempt in question under this peculiar variation, which has come under my knowledge, has been a case under intermittent. As relates to the desire, the simple fact is, that the patient feels a species of antipathy against some peculiar part of his body, added to the general disordered feeling, or he longs to commit the act by wounding that particular point; while, whether his aberration amounts to the desire of suicide or not, this very point or place is the one eternally forcing itself on his imagination as an object of hatred and revenge. And so perfectly insane is this feeling, that I have been informed by more than one patient who has suffered from it, that there is no conviction at the same time that death would follow; or rather, that the impression is as if the offending part could be exterminated or cured by the injury, and that the patient would then be well. And that suicide has actually been committed under this particular aberration, is well known from the more curious records of physic; while I need not do more than suggest one peculiar part of the body which has been often the offending and selected point; the act having been sometimes also, but not always, followed by death." 251.

Dr. M., we have no doubt, alludes to those cases of monomania, where men have severed the genital organs, from some momentary impulse of local antipathy or feeling which can only be known to the individual at the time.

"The philosophy of this, as far as my opportunities of observation have gone, and on the indicated ground of explanation, for these cases at least, is, that while the irritable, or jointly despairing and raging or angry state of this chronic fever is present, there is also a particular part of the body affected by an uneasy but undefinable sensation, such that the mind constantly reverts to it as a source of suffering. And if this local affection is not a neuralgia, or a condition of absolute pain, yet it is a local and nervous one of an analogous nature, always returning to that one point under the same stage of the fever or delirium. When, as is not unusual, it is seated in the head, it is even distinguishable by a dull pain, or a confusion, or a sense of 'buzzing' (for thus it is described by patients,) in one fixed place, indicating pretty clearly its real nature: while, in that particular case, I have the assurance of such patients, that the suicidal desire is exclusively directed to that individual spot, and that while a pistol would be the only acceptable mode, there would also be no satisfaction unless that were directed to this actual and only point. But I will cease, and allow physicians to exert on this solution of no easy question, the ingenuity which has not hitherto succeeded in producing an intelligible explanation." 253.

Our author inclines to the opinion of Strack, that an intermittent fever may consist of only one paroxysm, finding its natural termination after one attack—"and consequently that the fever called ephemera is, in reality, often a single intermittent." Strack observes, that this is the disease which terminates in an eruption on the lips. If Hippocrates is any authority on such a nice point, he may be thrown into the scale with Strack and our present author.

It was, and perhaps still is an opinion, (medical as well as popular) that agues, at least those of Spring, are salutary. This notion was entertained by Boerhave, and by many great names in our own country! Dr. M. thinks it probable that this idea arose from the fact that ague sometimes removes a chronic disease—or from another fact, that men not unfrequently become fat after a sharp attack of intermittent fever, especially in Flanders. It is curious that this obesity is not prevented by enlarged spleens and other visceral derangements, in many instances;—nay, even the daily paroxysms of ague, if not very violent, are sometimes compatible with an increase of corpulency. In whatever way we may account for this, the fact proves that ague, under ordinary circumstances, is not very prejudicial to the constitution, unless it be protracted or severe. But, on the other hand, we often see rapid emaciation, great loss of muscular power, and visceral derangement superinduced on intermittent fever, in all, but especially in hot and unhealthy climates.

### Anomalous, Obscure, and Simulating Intermittents.

Hitherto our author forbore to notice those epidemics, where very marked local inflammations of particular organs were such prominent features as to render the mere fever of comparatively little moment. He reserved them for this place, in order that he might the better illustrate the anomalous, localized, or simulating intermittents, a knowledge of which he considers as of paramount importance. These local inflammations affect almost every structure in the body, from the muscles and ligaments to the most vital organs—simulating or nearly equalling genuine idiopathic inflammations of these several parts, and introducing great diversity of opinion among practitioners as to the nature of the disease and the proper mode of treatment. If these phlegmasiæ occur, and all must acknowledge that they do occur, in marsh or continuous fevers, so it is reasonable to believe that they are attendants on intermittents, which are only kinds of the same fever, and thus they may be productive of great error or delusion.

"The inflammatory, or local affections, be they what they may, may be slender and truly supplementary; but they may also prevail so far above the fever, as in remittent, that they may appear to be a distinct disease, or the superior one; while if, further, the local affection should be permanent or continuous, when the fever has its intermissions, and perhaps long ones, as in quartan, and while, still further, that fever may not be very conspicuous compared to the local symptoms, it is easy to see that erroneous views and erroneous practice may follow." 280.

Dr. M. apologises for dedicating so large a space in his work to an investigation of the chronic forms of anomalous and simulating intermittents; but we think he need not fear censure on this account, for we verily believe this chapter to be one of the most original and valuable in the whole work. We regret indeed, that the tedious tautological *manner* in which he treats his subject, will deter most of the reading practitioners (not a very large proportion) of the day, from profiting by his researches:—since few of them will be at the pains of reading a passage twice, if they do not fully comprehend it at the first glance.

The first subject of investigation is the apoplectic state which sometimes commences the attack of intermittent as well as remittent.

"This state is not limited, either to the first attack or to the acuter forms of these fevers, although in Italy that appears to be the most common mode of its occurrence; as I have seen, in this country, a perfect apoplexy, to the eye, in all its characters, sufficient to deceive both the attending practitioner and the friends, lasting for eight hours, and occurring in a chronic tertian of many years standing, as a substitute for the cold fit." 287.

The danger that may result in practice, from an error here, is obvious enough. Venesection, to the amount generally employed on such occasions, might occasion palsy or even death—at all events, it would aggravate the symptoms. Dr. M. has seen local and limited paralytic affections produced by this practice, in many instances, "and in more than one, a perfect and incurable hemiplegia." We doubt whether Dr. M. could positively determine that a hemiplegia was thus produced. In common hemiplegia, permanent hemiplegia, there is generally an effused clot of blood; and if so, how can it be positively ascertained whether or not there is extravasation in consequence of venesection unnecessarily employed, without the aid of dissection? By this we do not mean to impugn the doctrine of simulated apoplexy. At page 455 of the 8th volume of this series, we have stated a very remarkable instance in our practice, and we could adduce several others; but we think Dr. M. has carried doctrine too far in the above cases of hemiplegia. We could more easily conceive that death would ensue from copious venesection in simulated apoplexy than that permanent hemiplegia should ensue.

"A modified condition of this nature, is the lethargic or comatose state, which occurs in the chronic intermittents as well as in the remittent of that character, and which also sometimes ushers in the first attack of an intermittent, in the same manner as the more perfect apoplexy does. It is incumbent on the practitioner to investigate this symptom or condition where it occurs, much more accurately than it is the custom to do; while it is abundantly easy to discover whether it belongs to this disease or not. To view it as an independent disorder, and as arising, according to the popular and fashionable error, from what is called a flow of blood to the head, is a most unpardonable mistake, from its leading similarly to injurious practice; to bloodletting and to cupping: the consequences of which also are, sometimes, paralytic affections, at others, slight epileptic ones, or even more decided fits of that disease; in others again, a modified fatuity, or a diminution of the intellectual powers, or a condition little short of absolute idiotism: and, even, in the least evil event, a long train of debility and nervous symptoms, with the further frequent consequence, as in the former case, of rendering chronic a disease which would otherwise have terminated by itself, or of protracting much longer and more severely, a disorder already chronic. It is one of the cases, and one of the modes of practice, yet but one out of many, which so often causes medical interference to aggravate the diseases of this nature. I shall only further remark, that this error is most generally committed, as I have seen in numerous instances, and as perhaps might have been anticipated, when the patient is corpulent, or of a certain form, or advanced in life, or when suspected of indulgence in eating and drinking: when it will be fortunate if he escapes bloodletting or cupping, to be simply deprived of the use of wine, or restricted in diet; though even practice of this moderate nature is not always without its bad effects." 289.

Paralytic affections, as occurring in these masked intermittents, are next adverted to by Dr. M. Dr. M. has not found any practitioners "who seem to know that palsy is the frequent produce of malaria, or the substitute for intermittent fever, or a symptom in that disorder." We believe that such cases are not generally known or suspected by British practitioners; but it has long been known that local paralysis, and that too of a very indomitable nature, is a common effect of what is called in hot climates, especially India, "*a stroke of the land-wind.*" Thus a soldier or sailor falls asleep, with some part of the body exposed to the night or land-breeze charged with miasmata from a jungly or marshy district;—and he awakes with the part completely paralytic, never more perhaps to be capable of sense or motion. This is a fact of frequent occurrence, and is strongly confirmative of our author's views.

"Apparently as a substitute for, or modification of, apoplexy or coma, however obscure the exact cause may be in either case, the first attack of an intermittent is sometimes a numbness, or a more perfect paralysis, varying in its extent as well as its severity, so as to affect only a few muscles, in different parts, or else to produce an absolute hemiplegia; which, however, may also be slight as well as complete, or may consist in a numbness or loss of feeling to that extent, without depriving the patient of his command over the muscles. Should the truth of such a cause for palsy be denied on the ground that we cannot explain its action, it must be recollected that it is no more a mystery than the production of apoplexy in the same cases; while the possibility is confirmed by the numerous instances of the same nature which occur, limited to single, and sometimes very small nerves, and either original, or succeeding to painful conditions of those. Every thing indeed tends to show, that if the diseases produced by Malaria are not, purely, and all, mere affections of the nervous system, the principal action of this poison is on that system, and the greater proportion of the disorders which are caused by it, disorders of the nerves. Whether the paraplegia so often occurring from exposure to cold, and especially among soldiers in bivouacs, is sometimes, or ever, a disorder of this nature, is a question that I cannot answer from such evidence as I have been able to procure, however this may be suspected in some cases." 292.

If these paralytic affections sometimes usher in a new intermittent disease, being, like coma, a substitute for the cold stage, so has our author seen them occur in the chronic form, and both under circumstances that could admit of no doubt: Thus, in a situation exposed to malaria, and never free from its diseases, in one or other form, a whole family, with the exception of two members, had ague, while these two were attacked with a paralytic affection suddenly—one in the leg and thigh—the other in the arm. In both these individuals, the paralytic affections as suddenly disappeared on the evolution of a regular quotidian ague. The practical error here is the same as in the simulated apoplexy—"the imagining a *flow of blood to the head*—and palsy to be treated by bleeding and evacuants." The result, our author observes, is, in severe attacks, to render that permanent which would only have been temporary, had the doctor not interfered, or had proper means been employed! Of such cases, he says he could state a great number from his own observation. The previous history of the patient, his diseases, his residence, and his treatment, left no doubt in his mind. Dr. M. mentions a few cases from the practice of others, stating the mere facts, and leaving it for the reader to draw his own conclusions.

Two sisters in one family, about the age of 30, were strongly marked with that complexion and physiognomy attendant on splenic disease, and were subject to those strange and anomalous symptoms which spring from chronic intermittents. They had spent a large portion of their time in one of the most pernicious districts of England, and the frequent occurrence of "spasms" led to bleeding in both cases. This was followed by more nervous symptoms, and led, of course, to more bleeding and purging. "The result, in one of these patients, was hemiplegia, and in the other paraplegia—and further bleeding being resorted to for these disorders, both of them died."

The other case was that of an officer, who is supposed to have had an intermittent fever at some previous epoch. "An inexplicable palsy of one limb was here the first occurrence, and, in a man of a constitution and time of life when the usual vulgar cause could not be suspected. Blood-letting was followed by palsy of the other leg; and, on repeating it successively, both arms, one after the other, became similarly affected, so that the patient at length became and remained almost an immoveable carcase." The final issue of this melancholy case is not yet known. Cases are quoted from Keratry and

Etmulla bearing upon this point, and then the following is introduced, not very flattering to the "healing art."

"In this example, an officer, a young man, who had suffered from the remittent fever in Spain, was suddenly seized on an English parade with a fit, or what is commonly called such, which was considered as apoplexy; being in reality a return of his intermittent. Being placed under the usual discipline, he was rendered partially paralytic, and at length, under two years of the general routine, became the mere shadow of a man, while previously most robust; losing also, with his strength, the better part of his intellect. Determined at length to join his regiment in the West Indies, he embarked in a transport, where, from there being no surgeon, all medical practices were suspended; from which moment he began to recover, and concluding just what I have here concluded, was in a few months restored to perfect health." 298.

This consequence of the malarious impression (paralysis) is generally admitted by those continental writers who have practised in unhealthy localities, but they have overlooked the share which bleeding and other evacuations may have had in the production or aggravation of the paralytic affection. Dr. M. assures us that his own experience on this point is wide and ample—and that it is far too precise to permit him to surrender his own judgment to the authority of others.

"Of partial palsies in the face, and of more general ones in the legs and in the arms, traced by the patients to cold, and, under my own views, appertaining to intermittent, I have known many instances rendered complete or incurable by bloodletting, when, from my own experience in similar ones, I have reason to believe that they would have passed away had the physician not interfered." 300.

Dr. M. takes occasion here to criticise pretty sharply some recent writers on palsy, for not even noticing, as one of its causes, the impression of malaria, or its consequence intermittent.

"And when I recur to a long series of observation, much more on the practice of others, of course, than in my own, I find such a mass of cases where it was to be often proved, very often suspected, that the paralytic affections arose from this very cause, that I am compelled to conclude that, in the first place, the cause in question is a very general cause of palsy, and, secondly, that the error of practice arising out of a wrong theory and the common one, is the source of the far greater number of incurable palsies daily met with in society; the increase of which also has been notoriously great for some time past, while it bids fair to proceed in augmentation, as the diseases of Malaria, from whatever cause arising, are themselves increasing, and as the improper practice to which I have alluded becomes also daily more prevalent." 302.

Besides these more sudden and palpable paralytic affections attendant on masked or open ague, there are others of a more gradual nature, which it is necessary to point out. These, perhaps, will be best understood by the statement of a case. This was an instance of a relapsing or chronic quotidian, where no previous local affection had existed. During the relapses in question, which generally lasted six or eight weeks, there came on gradually a feeling of weakness in one leg and foot, which increased so much during the disorder, that the patient could hardly put that foot to the ground.

"And although there was no pain or Neuralgia, it was easy to trace with the finger the course of the fibular nerve from the middle of the leg into the foot; as the slightest passage of the finger over it was attended by the well known tingling sensation produced by a pressed nerve; while a stronger pressure where it is nearest the surface, gave the equally well-known shock produced by striking the elbow, in another superficial nerve. It was obvious that the muscles chiefly affected in this case, were those to which this nerve principally belonged, and therefore that it was in a diseased state; that derangement appearing to consist in a diminution of its energy or power, added to an increased or morbid sensibility." 303.

The analogy of this to the proper neuralgia is, Dr. M. thinks, evident; and it must, in his mind, be considered as a modified degree of this malady. In subsequent relapses, the affection of the nerve was exasperated, and, ultimately, the ulnar nerve of each arm became similarly affected, with the same inability to extend the arms, without immediately feeling the tingling, as of a compressed nerve, along the whole course, from the arm-pit to the fingers. Dr. M. thinks that this affection of the nerve, whatever its true nature, must have been strictly local, or what might be termed anatomical—that is, appreciable by dissection. In the case now related, the nerves of the arms recovered, but those of the legs remained disordered for many years afterwards.

Dr. M. proceeds to quote cases from other writers, which, whether designed or not, support the doctrine which he is advocating respecting the connexion between palsy and intermittent. Thus, Sauvages quotes a case from Chaptal, under the very term tertiana hemiplegica, which Dr. M. thinks might have opened his eyes to the connexion in question; but it did not. In another case, quoted by the same learned nosologist, the paralytic affection came on every day, and disappeared with the accession of the proper quotidian.

"Palsy in the form of paraplegia, of a periodical and quotidian intermitting character, is also described by Torti, from Chaptal. Where the same writer, from the same authority, quotes a similarly periodical hemiplegia absolutely perfect, while it was quotidian and intermitting, lasting also for ten hours, he remarks that it was always increased by purging as well as by bloodletting, and that on changing the practice, it was cured by bark in nine days. This particular case is of value, as a warning to practitioners; and, from the nature of the attending and preceding symptoms, perhaps of somewhat more value than common." 310.

A case from Morgagni is still more remarkable. In this, one side was permanently paralytic, while the other was attacked every evening with a palsy, which disappeared in the morning—the patient, after seven or eight such fits, dying of peripneumony. Various irregular cases of this kind, where the paralysis was attended with convulsions, are noted by the same author. Neither Morgagni, Sauvages, nor any of the writers on palsy, however, appear to have suspected a connexion between intermittent and that disease. The connexion of apoplexy with intermittent has been largely noticed by Ramazini, who describes the apoplexy as occurring at all periods of the disease, as well as at its commencement. This connexion was also known to Morton, and its nature must have been appreciated, when he prescribes bark for the cure of the apoplexy, as well as the ague. Theon de la Chaume describes an epidemic tertian, accompanied by apoplexy, as prevailing at Ajaccio, in two different years, 1773-8. The same has been often seen in Bresse, the most pestiferous part of the Lyonnais.

"It is a noted fact, that it is the effect of chronic or habitual intermittents to injure or destroy the intellectual faculties, as I had occasion to point out already when treating of remittents. This is notorious in the countries where these disorders prevail, and very remarkably, as I formerly said, in the Maremma of Tuscany, where even absolute idiotism from this cause is common; the fact being marked, even to cursory travellers, by that apathy, listlessness, or indolence of mind, gradually approaching to fatuity, which I formerly described. If the cause be obscure, it cannot well be more obscure than every thing else which belongs to the action of Malaria; while the fact of the universal influence of this poison on the nervous system, local as well as general, leaves no difficulty, at least in believing that it may produce such effects on the mind." 322.

But ague, for a long period of its existence, only affects the mind with irritability, or increased sensibility, as evinced by peevishness, exaggerated views of evil, increased sensibility to bodily suffering—to say nothing of nervous sensations beyond number. In process of time, however, a train of opposite effects come to prevail. The mind becomes, as it were, torpid, as evinced by listlessness, and submission to present evils, with scarcely a wish to escape them. This is a striking feature in the inhabitants of pestiferous countries. Dr. M. relates some curious instances where, in people who had laboured under this disease, the organs of sense lost their aptitude for pleasurable sensations, "their complaints being, that beautiful objects, such as pictures, natural scenery, &c. which, before that, or when in health, had been most pleasurable or engaging, seemed to make no impression at all on the sense."

"From such patients I have received also the same complaints and statements, with respect to the other usual causes of simple pleasurable feelings; and very particularly from those who, as musicians, were accustomed to delight in music, not less from science than feeling; those being, that they seemed to suffer under a positive insensibility as to what used to be a source of the most refined delight, although labouring under no affection of the temper, nor any of those sensations commonly called hypochondriacal. And thus have others complained that the most grateful odours had ceased to give pleasure, that the scent of a rose was not only powerless, but produced absolute pain by reminding them of what it once was; while every attempt to revive the former associations connected with this and other similar objects of delight, was unavailing." 325.

This *mutatis mutandis*, Dr. M thinks, is precisely the progress in those cases where single nerves are affected, instead of the whole cerebral system. "In the neuralgia, the first action of the cause is an increase of sensibility, reaching to the highest imaginable degree of pain;" but the progress of this is to palsy—"or the excessive sensibility is succeeded by a diminished one," as if the sensibility were exhausted by the previous over-excitement.

After many ingenious and interesting observations on *periodical* mania, vomiting, hysteria, palpitation, and other irregular modes or forms of intermittent, our author comes to the occasional connexion of rheumatism with malarious diseases.

Dr. M. without attempting to unravel the difficult pathology of this disease, trusts that he will be able to prove that there are cases which belong to intermittent—"cases bearing an analogy to certain modes of neuralgia, and possibly differing from it, by the affections being seated in the numerous and minute ramifications of a nerve, instead of attacking a trunk, or a leading branch."

"The most simple case of all, while it is one that ought never to be mistaken, is that where a rheumatic pain in some particular muscle is strictly periodical, returning and ceasing in regular paroxysms. In such cases, the part affected may sometimes be exceedingly limited, occupying only a few fibres of a muscle, though, even then, the pain is often severe; while in others, the extent may be very considerable. Thus even the whole body may suffer under it; or rather there may be so many different muscles affected, in some place or other, that scarcely any movement can be made in which some one or more of the disordered portions is not brought into action; conveying thus, to the patient, the feelings as of an universal rheumatism." 369.

Such a periodical recurrence, he observes, might satisfy a practitioner respecting the true nature of the disease; but it will often be attended with other symptoms, explanatory of its cause. Thus, it will be found to occur in persons who have been previously affected by intermittents, forming in itself a period of relapse, and a substitute for the more common modes of chronic disease. In other cases, the rheumatic pains will alternate with some of the other marked symptoms belonging to this disease:—or it may cease on the appearance of the common symptoms of intermittent.

Dr. M. tells us that there is a certain *physiognomy of the cold stage*, however wanting the actual feeling of cold may be, which is never absent at some period or other of this, and of all other malarious diseases, and which has, on endless occasions, enabled him to pronounce, from the first sight of the patient, on the nature of the disorder to which he has been summoned, with the assurance of its mysterious nature by the medical attendant. The power of distinguishing this physiognomy, however, requires the TACTUS ERUDITUS of the experienced practitioner.

The kind of rheumatism here alluded to, as connected with intermittent, will, of course, be generally of the chronic kind, and our author does not attempt to say what proportion they bear to the common rheumatism, dependent on other causes; but he thinks it probable that they form a considerable proportion of what pass under the name of chronic rheumatism.

"The more serious question remains; whether that which is esteemed acute rheumatism, a disorder too well defined and too familiar to require description here, may be a mode of intermittent. I do not mean to suggest at present, that every acute rheumatism is a disorder belonging to this class of diseases, or that, as in the chronic variety, there are not cases which are independent disorders, or affections generically different, although it seems to me, that even this is a question far from decided the other way. The question at present is, whether there are not acute rheumatisms of the most regular form, which are truly modes of the quotidian intermittent, or of the remittent, possibly, originating in the same causes: and if it shall be decided that this is the fact, and that there is also an acute rheumatism generically different, then we shall probably be able to explain the causes of the contests so long maintained respecting the use of bark in this disease.

"The facts which would seem to prove this opinion are chiefly these. There is a periodical exacerbation, if there is not always an absolute remission of the pains; and the duration of the disease is very analogous to that of a remittent, or of one period of an intermittent. The causes correspond, if they are not identical, while the remedy is often the same; since, after all that has been disputed, there is no doubt that many cases are cured by bark, and that blood-letting is not only often ineffectual, but pernicious; its action altogether, being, in fact, very similar to that which it exerts on remitting and severe intermitting fevers." 375.

Thus, while in acute rheumatism, the misapplication or abuse of blood-letting often produces the chronic disease; so, a similar practice frequently induces the chronic state of intermittent—or converts an acute and terminable case into a durable one.

"It is not impossible also that the termination of the pains of acute rheumatism, succeeded by affection of the brain, and so often producing death, may be an analogy to what happens in other cases of intermittent diseases, where one local affection is exchanged for another, or disappears, to be replaced by an augmentation of the general fever." 376.

We are so far outstripping our limits that we must pass over our author's investigation of those cases of pleuritic and catarrhal affections, anginæ, hepatitis, gastric and splenic disorders, ophthalmia, &c. which occasionally assume a remittent form, and depend on a malarious cause. Every man must have repeatedly seen rheumatism of the intercostal muscles mistaken for pleurisy, and venesection injuriously, or at least injudiciously resorted to for its cure.

"If I have seen constitutions utterly ruined by a perseverance in this wrong practice, if I have seen patients condemned to believe themselves labouring under consumption in these cases, with all the expensive and vexatious consequences that follow such an error, there are doubtless many physicians to whom the same facts have occurred." 380.

Yet it is by no means difficult to distinguish between the internal and external complaint, even without the aid of percussion or auscultation. The following case is interesting, and we shall introduce it here.

"The patient was a young man in the higher rank of life, and the pain in the side was termed pleurisy, though no cough was present, and very little fever; so little, that not even confinement to bed was necessary. Blood-letting was resorted to, very actively, and was followed by increase of the pains; and, not to prolong a tedious history, these pains continued or returned occasionally, during nearly a whole year, while, during all that time, this remedy was repeated, often, many times in a week. If it was plain that this, by merely negative reasoning, must have been a rheumatic disorder, there was even much plainer evidence, in the periodical returns of the pain, after some weeks, that it was also the intermitting disease; while the physiognomy and appearance marked, once in every day, a decided cold stage. Still further, after about five months, there came on a pain in the shin-bone of one leg, regularly periodical, and lasting five hours; during which, the rheumatic pains among the ribs diminished or ceased, yet without leading the physicians to a correct judgment of this case, as it ought to have done; being a true neuralgia, interchanging partially with the original intermitting rheumatism." 382.

This case was considered to be a wonderful and mysterious one, and it was proposed to make an incision through the periosteum, on a supposition that the bone was diseased. The patient's health was greatly reduced, and he did not recover strength for many years, when the disease terminated in a regular ague that has harrassed him ever since in a chronic form!

Among other intermittent rheumatisms, Dr. M. notices lumbago, which is, at least sometimes, a modification of the class now under discussion. One case is mentioned where, what was called a regular lumbago, and treated as such, "was suddenly and spontaneously removed, and immediately succeeded by the common neuralgia of the face." A similar case is recorded by Dr. Pearson. Dr. M. considers those pains situated in non-muscular parts, as improperly denominated rheumatism. They belong to the neuralgiæ, as those of the face and head.

> "There is something singularly periodical in the attacks of a catarrh which often comes on in summer, and, as it would appear, most commonly from exposure, not simply to heat it would generally seem, but to heat where vegetation is present. This well-known disorder is produced by hot-houses or green-houses; and, in the public estimation, it is particularly caused by hay-fields. Hence the term hay-fever, lately become fashionable." 394.

Dr. M. has not much experience of this disorder, but he knows that it is one which is aggravated by the remedies which aggravate intermittents—namely, blood-letting and other evacuations. Dr. Bostock, who appears to have suffered from this complaint in person, has given a full account of it.

We must pass over a great many complaints of a periodical character, and which are not improperly classed among those dependent on a malarious origin, in order that we may dedicate a few pages to therapeutics.

### Treatment of Intermittents.

Dr. M. does not pretend, of course, to offer any new cure for this class of complaints; but he has dedicated a large space to comments on those modes of treatment which have been employed by others. He observes, that the remedies which will cure a recent intermittent, will be much less efficacious in one of long duration or relapse—but he does not conceive that any distinction is necessary as to the types of intermittent maladies—"since, in all of these, the remedies are the same," requiring only such modifications as the obvious circumstances of the case may indicate.

The simplest remedies are those which act on or through the mind, and—"their number is far greater than I choose here to record." Their action is undoubted—but they are best suited to new attacks of new diseases. There is no evidence of their efficacy in remittents, and it is curious that remedies of this class have been more successful in stopping tertian than quotidian agues. Without *faith* in their power, they are nugatory —and this explains the rationale of their operation in most cases, in conjunction with disgust, fear, and other strong mental impressions. Under this head (Dr. M. thinks) must be classed "a vast catalogue of internal medicines of the most discordant properties or of no properties at all." He is of opinion that bark itself sometimes acts in this way—namely, by *confidence.*—Spiders' webs may act by disgust or horror, and so on.

The next class of remedies includes those which make a powerful impression on the system, and especially on the stomach, immediately before or soon after the commencement of the fit—by which the paroxysm is prevented or abridged. Alcohol, opium, spices, are the basis of this class. Those medicines which we term tonic, as bark and arsenic, are next noticed by our author. He cannot conceive why they should have the term *tonic* applied to them, "so unphilosophically lax are the ideas attached to that term." After some general observations on the management of the three stages of the paroxysms, Dr. M. comes to make some comments on the administration of particular remedies, beginning with the bark. Between the foreign practice of giving as large a quantity of bark as can be taken, within one, two, or more intervals, and then ceasing for a time, and the English method of giving the medicine in less quantities, "persevering without limit," Dr. M. finds it difficult to decide, as to the comparative advantages.

"But while I must return to this question immediately, one remark seems well founded, however, often neglected; and it is, that the perseverance in bark beyond a few days is nearly useless; while, if it has been said that whenever it offends the stomach, it produces no good effect, this is contradicted, as I shall presently show, by other physicians. And further, it seems often true, while even less known, that where a large dose is inefficacious, a small one is often useful; or, in reality, that ten or fifteen grains will sometimes produce a better effect than a drachm. Of the various preparations, the now common combination of kina, its sulphate, seems the only one which deserves a preference to the bark in substance, while it will probably prove to be in every instance preferable." 445.

Dr. M. next gives the opinions of various authors as to bark; but these we need not enumerate, nor even notice. We shall stop for a few minutes, however, on the subject of arsenic, a remedy that "has been lauded beyond its merits, and often also condemned and shunned, rather from the fear excited by its name than any thing else." The following sentiments coincide very nearly with our own experience.

"I have little, therefore, but my own experience to judge from; and this is, in the first place, that it is less efficacious than bark in diseases of a highly febrile character, and of long duration; or that as the intermittent approaches nearer to the remittent, arsenic becomes an uncertain remedy, and that in the very chronic disease it appears to me to possess no power at all; though I know not, that, in these latter cases, it is more nugatory than any other remedy. In a new and a very simple intermittent, and in the tertian particularly, it seems to offer a more rapid remedy than bark, while its superior convenience is manifest.

"But if I were to compare it with bark in those cases where the disease puts on the anomalous symptoms or characters which I have described, I should often judge it a more effectual remedy than that; and although my own experience is far from sufficient to decide this point, I have also found it the best medicine in all the cases of the most purely local affections, or in the neuralgia; not but what it fails much too often, even in these, and particularly where they are of long standing." 452.

Dr. M. objects to the form in common use, Fowler's solution, and assures us that the common white arsenic, in powder, has succeeded, when the solution has failed. The sixteenth part of a grain is that which Dr. M. has employed, repeated three or four times a day, rubbed down with lump sugar.

"With respect to the superiority of arsenic in substance to its neutral salt, I may quote the experience of a friend, who, residing in a district where tic douloureux is extremely common, and where the solution seldom succeeded, now reports to me that he finds the powder almost infallible; giving it without the least inconvenience to the extent of 1-12th of a grain for a dose, and finding that its utmost limit is 1-8th, which can seldom however be endured, though having administered 1-6th without further evil consequences than gripings." 453.

This information is important, if true; but the apprehension of mistakes in the shops will prevent many from giving the common arsenic a fair trial.

"As this remedy is held to be attended with danger, and also with ultimate bad consequences, I must here bestow a few words on that subject. When given in excess, short of its properly poisonous effects, the symptoms are various, but the following have been observed; headach, sweating, tremors, nausea, vomiting, griping pains, with spasms of the lower extremities, and, sometimes, affections of the urinary passages; more frequently a red eruption on the skin, with swellings about the eyes and other parts, resembling that produced in what is called a surfeit, from eating muscles, and, in particular persons, many other substances. I must also remark, that, as in this latter case, there are individuals who thus suffer from it, even in the minutest doses, and that the eruption of the skin appears to be one of the most common effects, generally however limited to the face and the breast. That effect, together with slight nausea, are the ordinary and commonly the sole ones, unless the dose be excessive." 454.

Dr. M. has never known any ulterior or permanent ill-consequences arise from the use of arsenic. "They are all easily removed by brandy, as is the common surfeit, or by opium; and if not, they cease of themselves in a few hours."

In some constitutions, and after a few days' exhibition of the arsenic, the pulse becomes quickened, and the skin hot and dry; while there is that peculiar feeling of languor and debility that is known to result from mercury. Flatulence and sense of distention very often attend this condition. In such cases it is prudent to desist, though our author has not seen any bad consequences result further than the above. He does not believe that paralysis has ever been produced by this medicine.

On the cold bath, mercury, and purgatives, Dr. M. makes some cursory remarks, which we pass over. They are, generally speaking, condemnatory—excepting when the above means are used with great caution. Against purgation in particular, Dr. M. entertains considerable aversion.

"But in the chronic varieties, and in these, in proportion to their duration and the debility of the patient, while it is not less indispensable to maintain the bowels in a natural state, actual purging is almost invariably pernicious, unless applied to for accidental and specific purposes, of which every physician can judge. The common, the very common effect of it, is to cause relapses or returns of a disorder that has ceased, and thus to render chronic a case that might have terminated; and when what are called courses of purging medicines have been resorted to, whether from any theory of their utility, or from a mistaken view of the symptoms and their cause, it is not unusual to see produced, the most inveterate cases of chronic intermittent, and very generally also to find them under some anomalous form that might never else have occurred." 460.

Of the truth of these observations we have seen some very curious and melancholy illustrations during the last eighteen months, a period remarkable for the prevalence of intermittent diseases under various forms. The same observations, Dr. M. adds, apply to all the cases of neuralgia under its endless modifications. Speaking of the pernicious effects of active purgation now employed in all disorders, chronic as well as acute, Dr. M. makes the following sarcastic reflection.

"If the united ignorance and presumption of self-empirics could ever find an excuse, they might indeed claim it in this case; when they see practitioners of high fame, if notoriety be fame, following similar universal systems of cure, applying salts or 'the blue pill' to every disorder or symptom in the nosology, and without inquiry; and thus, while saving themselves all the trouble of thinking, rendering physic an art which may be practised by any one, without previous study or present observation; since the Alkahest does all.

"If it is strong language, it is scarcely exaggerated to say, that this universal tampering with salts and calomel is one of the greatest misfortunes which fashion and folly united ever entailed on England; while it is even matter for satirists, to find that a course of the waters of Cheltenham or Leamington, at once powerful and precarious, a system of active practice which can never be neutral, and which if not useful must be pernicious, is held a fashionable necessity, a mode of passing time, equivalent to any other expensive system of idleness on which society has stamped a certain reputation. But this is a small portion indeed of the evil, when we review the whole of this most extraordinary fashion, in a manner however in which I cannot undertake to examine it here. Whether the old Roman practice of emetics was more or less pernicious than that of the dinner-pills or the morning salts, it is not here my business to inquire; but he is widely mistaken who imagines that the injury produced by frequent or habitual gluttony is to be repaired by the further injury resulting from frequent or habitual purgatives." 464.

Speaking of *physic*—(whether as a *drug*, a science, or a trade, we are not quite certain)—Dr. M. unequivocally accuses it of being the principal *cause* of our diseases!!

"Let that any family or any individual thus educated on purgatives, (provided indeed the health is not utterly ruined,) take but courage enough to destroy the medicine-chest and *lock the door against the physician*, and they will soon find which was the cause and which the consequence." 464.

Dr. M. however, admits that there are some other causes of disease besides physic—for example, malaria, idleness, luxury, peculiar modes of life.

"I must also (says he) notice, as perhaps the greatest and most general cause of nervous affections, particularly in men, *a state of things which seems to have been very much overlooked by those physicians who have speculated on this subject.* I allude to the great increase of mental employment, or of study and business or occupation, requiring mental rather than bodily exertion, connected also with that which frequently becomes a species of disease in itself, EDUCATION, or study and talents, and the latter habitually exerted—added also to confinement and all its collateral evils, and further, too often accompanied by that anxiety, with its occasional attendants or sequels, disappointment, which is the produce of the especial ambition, either as to wealth, or honours, or fame, which denotes the present times." 467.

Still, all these are inadequate, Dr. M. thinks, to the production of that wide prevalence of dyspepsia which characterises the present race of English. Purgation, he conceives, is the other grand item in the etiology of bilious, nervous, and dyspeptic disorders.

From what has preceded, we need hardly remark that Dr. M. is a decided enemy to venesection in intermittents, except under the most rigid restrictions. In the hot fit it is rarely necessary—in the intermission it is dangerous—and, we should imagine that Dr. Mackintosh's practice of bleeding in the *cold stage* must have given our worthy author an ague, considering how very susceptible he is to every morbific impression!

On the other hand, as might be expected, Dr. M. is a steady advocate for good wine and good living generally, in the class of diseases now under review. The partiality, indeed, with which Dr. M. seems to view good cheer, would induce us to believe that he has been so fortunate as to partake of the pleasures of the table, in those situations where the "feast of reason and the flow of soul" add not a little to the enjoyment of the "good creatures" of this world. We are by no means inclined to criticise his dietetics, however, where chronic intermittent diseases are to be managed. Depletion and starvation, in such cases, would be highly deleterious. On what principle, indeed, could we prescribe bark and arsenic, in conjunction with drastic purgatives, venesection, and low diet? The disease is one of debility and irritability—and in these cases, tonics, stimulants, and generous food and drink are called for.

The last subject which we shall notice is "change of air," the efficacy of which is undoubted in most chronic diseases, but in malarious diseases particularly. It is not merely the removal from a bad air to a good one that is productive of so much benefit. The operation, in this case, seems to be that of breaking the habit of the disease—as a chronic intermittent appears very often to be a mere habit.

"If this be the case, a difference in the quality of the air breathed, which is what the popular phrase would signify, is not in itself the remedy; though respecting this we really are not in a capacity to argue at present, since it is most certain that the atmosphere, in different states or places, produces effects on the body, of which our present chemistry does not enable us to investigate the causes. The lungs, or the organs here concerned, to whatever extent, are in reality chemical agents superior in discernment or power to those of our laboratories; or the involuntary and unconscious animal is that chemist which the reasoning one is not; carrying on operations which he can neither imitate nor discover, and detecting substances which he cannot find." 492.

Here we must conclude this article—an article which embraces half a volume, and on which we have expended more labour than our readers, or perhaps the author will give us credit for. Nothing but a strong conviction that the work before us contains a multitude of valuable gems, which readers in general will not take the trouble to pick out, could have induced us to bestow so much labour on a review, at this season of the year, (June 1828,) when the town is so full of men and malaria, that our literary labours only *commence* when "church-yards yawn," and all our other brethren (accoucheurs excepted) are fast asleep! In rendering Dr. Macculloch's work more accessible to the profession, by diffusing it in a portable and perusable shape, we are conscious that we are doing the state some service—and the author no injury. We have several other articles from the same work in reserve.

---

II.

*Surgical Observations on the Treatment of Chronic Inflammation in various Structures; particularly as exemplified in the Diseases of the Joints.* By JOHN SCOTT, Surgeon to the London Ophthalmic Infirmary; and Assistant Surgeon to the London Hospital. Octavo, pp. 291. 1828.

IT was our intention to have noticed this work at the time of its first appearance, and with that view we then perused and examined it with some attention. By some accident, however, the copy prepared for review was mislaid; and the subject in consequence was overlooked and forgotten, until chance again, within these few days, presented the stray volume to our hands. But it is now, we fear, too late to pursue our original design; for the work no longer possesses any interest on the score of novelty, nor are its intrinsic merits such as to entitle it at this late period to an extended examination here. Under these circumstances, we shall, on the present occasion, confine ourselves within very narrow limits; our object being now, not to analyse the work itself, or expatiate on the subjects of which it treats, but simply to discharge a duty we have long imposed upon ourselves, that namely, of placing on record in these pages every cotemporary statement or suggestion, which seems calculated to be of any practical utility in the art of medicine.

1. *On the Treatment of Ulcers on the Legs.*

It is well known to most of our readers that the mode of treatment introduced some years ago by Mr. Baynton, of Bristol, for the cure of ulcers on the legs, viz. by *adhesive straps*, has never been found so successful in the hands of others, as it was stated to have been originally in his. Occurrences of this kind, however, are too common in the practice of physic to excite much surprise, and too easily accounted for on general principles to demand much investigation. Mr. Baynton's mode of treatment, therefore, has gradually been suffered to fall almost into disuse; and practitioners in the present
